# Impacts of self-reported communication perception and shyness on the public speaking assessment of university students

**DOI:** 10.1590/2317-1782/20212021225en

**Published:** 2022-10-17

**Authors:** Daniel Lucas Picanço Marchand, Lucas Sávio Rodrigues Carvalho, Diego de Souza Leal, Sheila Gonçalves Câmara, Glaucya Madazio, Mara Behlau, Mauriceia Cassol

**Affiliations:** 1 Universidade Federal de Ciências da Saúde de Porto Alegre – UFCSPA - Porto Alegre (RS), Brasil.; 2 Centro de Estudos da Voz – CEV - São Paulo (SP), Brasil.; 3 Escola de Saúde Pública do Rio Grande do Sul – ESP/RS - Porto Alegre (RS), Brasil.

**Keywords:** Speech, Language and Hearing Sciences, Speech, Communication, Communication Barriers, Shyness, Persuasive Communication, Interpersonal Relations

## Abstract

**Purpose:**

To compare self-assessment when speaking in public, using the Self-Statements During Public Speaking scale, with the communicational perception and self-reported shyness of university students.

**Methods:**

This was a prospective cross-sectional observational study. University students from different areas of knowledge in Brazil were invited to participate in this study. Those who agreed to participate were included. Participants received an electronic invitation and filled out a form created on the Google Forms platform that contained sociodemographic questions, on self-perception as a good speaker, on ease of expression, on shyness, and the Self-Statements During Public Speaking scale. The means of the Self-Statements During Public Speaking scale were compared with the self-perception as a good speaker, the ease of expressing oneself, and shyness.

**Results:**

Participants who considered themselves to be good communicators, those who believed they had an ease to express themselves, and those who were not shy had better self-perception of their public speaking skills.

**Conclusion:**

positive communicational self-perception, as well as less shyness self-perception, are related to a more favorable self-assessment in relation to public presentations.

## INTRODUCTION

Communication permeates interpersonal relationships and depends not only on language skills but is also influenced by the psychological aspects of a person^(1)^. In the academic context, an important form of communication is oral presentations^(2)^ in the form of speech by university students, with their peers as an audience. This aptitude is increasingly required since in the future it will be crucial for these undergraduates in their different work environments^(3)^.

The fear of public speaking is considered a subtype of social anxiety. It affects a considerable portion of the population, with a high prevalence in university students^(4,5)^. It is usually associated with communication apprehension, characterized as a type of shyness related to the expectation of talking to people^(6)^. Both affect the ability to create and decipher messages, in addition to inciting a negative self-assessment, which can impact an individual's personal and professional spheres^(4,7)^.

The ability to speak well in public promotes both social satisfaction and a subject's self-esteem^(8)^. However, the concern about being judged, and comparing oneself with others, in addition to the anticipation of losses resulting from an unsatisfactory oral presentation makes the experience of speaking in public uncomfortable^(8)^. Thus, individuals with a lack of confidence, shyness, inadequate preparation, tension, fear of making mistakes, and poor speaking skills in the target language end up suffering from fear of speaking in public^(9)^. Shyness plays a special role in these difficulties in dealing with the situation of public speaking, as it is an anticipatory negative self-assessment. Shyness is considered a personality trait in which an individual makes negative assessments of themself, by creating discomfort or inhibitions in social situations. This can potentially produce barriers at work, friendships, emotional relationships, and leisure^(10,11)^.

To understand the causes and impacts of fear of public speaking in an individual, some protocols and scales were created^(12-14)^. One of these scales is the Self-Statements during Public Speaking – SSPS^(14)^, with cross-cultural adaptation^(15)^ and psychometric validation for Brazilian Portuguese^(16)^, which is now called the Escala para Autoavaliação ao Falar em Público. The purpose of the tool is to assess fearful thoughts during a presentation to an audience.

According to the literature, the emotional state influences communicational factors. Thus, the objective of this study is to compare the self-assessment when speaking in public, using the SSPS scale, with the communicational perception and self-reported shyness of university students.

## METHODS

### Study design

This is a prospective cross-sectional observational study approved by the Research Ethics Committee of the proposing institution under number 2.729.273. Due to the study design, the STROBE checklist was used to guide the writing.

### Participants

Undergraduate students attending higher education institutions in Brazil were invited to participate in the study. All participants who agreed to respond to the assessments and agreed to the provisions of the Free and Informed Consent Term were included. Those who were younger than 18 years of age or who provided incorrect and/or incomplete answers to the assessment instruments were excluded.

### Sample calculation

A pilot study was carried out using the responses of 30 randomly chosen participants. Based on this study and considering the heterogeneous student population in terms of responses (50%), sampling error of 5%, and confidence of 95%, the minimum number of responses for the sample to be representative of this population was estimated at 384. A response rate of 30% was expected - thus, it was necessary to invite at least 1280 students to respond to the survey.

### Data Collection

Participants received an invitation, via email or social networks, containing an electronic form built on the Google Forms platform. They were asked to answer general identification and communication questions, and the Self-Statements during Public Speaking scale (SSPS). The collection took place between October 2018 and June 2020.

#### Questionnaire on communication

Participants were asked to answer the following closed questions, created by the authors (Chart 1).

**Chart 1:** Questionnaire on communication

**Do you consider yourself a good communicator?**
*Understand as a good communicator: a person who can easily express their ideas verbally and pay attention to what is being said to them.*

() I definitely consider myself a good communicator
() Sometimes I can be a good communicator because I can express myself well. But occasionally I don't pay attention to the content of the message
() Sometimes I can be a good communicator because I pay attention to what I'm told. But occasionally I have trouble expressing myself
() I don't consider myself a good communicator
**When you need to communicate, you:**
() Can express yourself very easily, without the need to complement or repeat what was said
() Can express yourself with some difficulty, often needing to complement or repeat what was said
() Can express yourself with great difficulty, almost always needing to complement or repeat what has been said
() Cannot express yourself, always needing to complement or repeat what has been said
**As for shyness, do you consider yourself:**
() Not shy
() A little shy
() Very shy

#### Self-Statements during Public Speaking (SSPS)

The SSPS^(14-16)^ presents 10 questions that the participant must answer keeping in mind their perception in situations of public speaking. The answer key corresponds to a six-point Likert-type scale, with 0 being marked when the respondent totally disagrees with the statement and 5 when they totally agree. The scale has two subscales with five questions each: positive self-assessment (SSPS P), and negative self-assessment (SSPS N). The total score (SSPS T) is given by the sum of the scores of the 10 questions, with the scores for the statements of the negative self-assessment subscale being inverted. The final score of this scale is between 0 and 50 points and the interpretation of the result is: the higher the score, the more comfortable a subject feels when speaking in public.

### Sampling

**Figure 1:**
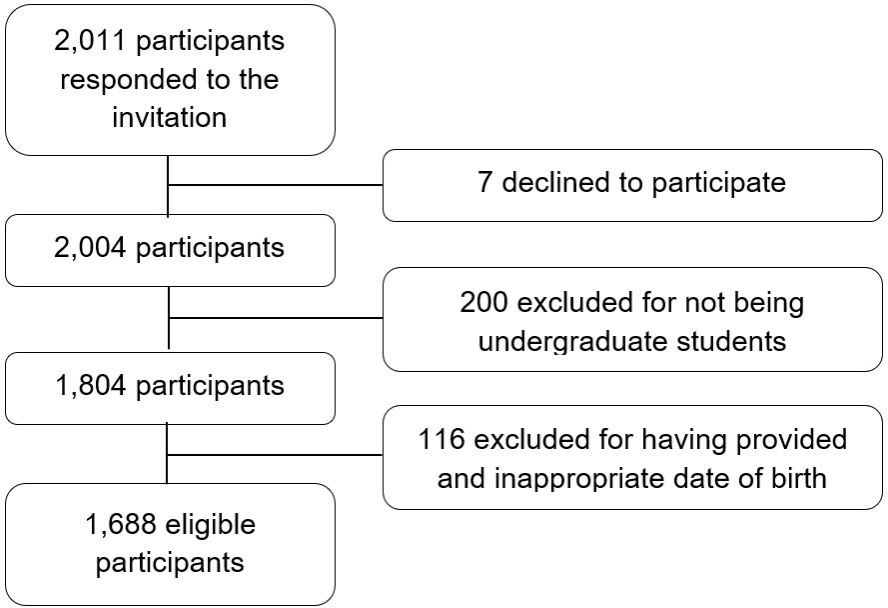
Sample composition flowchart

After applying the eligibility criteria (Figure 1), the final sample is composed of 1688 participants.

Table 1 shows the sample characteristics data. The sample is composed of participants aged between 18 and 61 years (24.00 and 5.74 - mean and standard deviation, respectively), mainly women (n=1103, 65.3%), caucasians (n=827, 49%), and public university students (n=1555, 92.1%). The area of knowledge of the participants and the geographic region showed no statistical difference concerning the variables analyzed in this study.

**Table 1:** Characteristics of the sample of university students (n=1688)

**Variable**		**Description**
Gender – n (%)	Male	585 (34.7)
	Female	1103 (65.3)
Age – $\bar{X}$ ± SD		24.00 ± 5.74
Ethnicity – n (%)	White	827 (49.0)
	Black	234 (13.9)
	Brown	584 (34.6)
	Yellow	37 (2.2)
	Indigenous	6 (0.4)
College type – n (%)	Public	1555 (92.1)
	Private	133 (7.9)
Region – n (%)	North	61 (3.6)
	Northeast	552 (32.7)
	Midwest	82 (4.9)
	Southeast	607 (36.0)
	South	383 (22.7)
	Another country	3 (0.1)
Area of knowledge – n (%)	Agrarian Sciences	177 (10.2)
	Biological Sciences	90 (5.3)
	Health Sciences	392 (23.2)
	Exact Sciences	218 (12.9)
	Human Sciences	172 (10.2)
	Social Sciences	420 (24.9)
	Engineering	158 (9.4)
	Linguistics	66 (3.9)
Undergraduate year– n (%)	First-year	305 (18.1)
	Second-year	406 (24.1)
	Third-year	345 (20.4)
	Fourth-year	380 (22.5)
	Fifth-year	208 (12.3)
	Sixth or higher	44 (2.6)
Income– n (%)	Less than 1 minimum wage (R$0.00 ~ R$953.99)	276 (16.4)
	From 1 to 3 minimum wages (R$954.00 ~ R$2861.99)	698 (41.4)
	From 3 to 6 minimum wages (R$2862.00 ~ R$5723.99)	393 (23.3)
	From 6 to 10 minimum wages (R$5724.00 ~ R$9539.99)	201 (11.9)
	More than 10 minimum wages (R$9540.00 or more)	120 (7.1)
Work– n (%)	No	972 (57.6)
	Yes	716 (42.4)

**Caption**: $\bar{X}$ = Mean; SD = Standard Deviation.

### Statistical treatment

The analysis of data distribution normality was assessed using the Kolmogorov-Smirnov test. Categorical variables were expressed as absolute and relative values, while quantitative variables were expressed as mean and standard deviation. One-way Analysis of Variance (ANOVA) was used to compare the scores on the SSPS scale with the other variables, and Tukey's post-hoc was used to identify in which categories there was significance. For the analysis of correlation between variables, Pearson's Correlation Coefficient was applied. A significance level of p≤0.05 was adopted. Statistical analyses were performed using the Statistical Package for Social Sciences (SPSS) software version 25.0 (IBM Corp. Released 2017. IBM SPSS Statistics for Windows. IBM Corp., Armonk, NY).

## RESULTS

### Perception as a communicator

Table 2 showed that the SSPS scores were different when comparing students who rated themselves as (1) Good communicators, those who (2) had difficulty in expressing themselves, those who (3) had difficulties in receiving information, and, still, those who (4) did not consider themselves good communicators. The results of the four groups were different, in SSPS T (p<0.001), SSPS P (p<0.001) as well as SSPS N (p<0.001). The sample means were 29.28 ± 10.50 for the SSPS T, 15.56 ± 5.08 for the SSPS P and 13.72 ± 6.89 for the SSPS N. The sample had, above all, students who considered they had difficulties in expressing content (n=861, 51%). Good communicators scored higher, that is, they feel more comfortable speaking in public. They are followed by those who have difficulty in reception, then in expression, and, finally, those who do not consider themselves good communicators.

**Table 2:** Comparisons between the SSPS subscales and self-perception as a good communicator in university students (n=1688)

	**Self-perception as a good communicator**	**n (%)**	**Mean**	**SD**	**Relationship with another level**	**SE**	**p-value**
SSPS T	Not consider a good communicator	391 (23.16)	22.03	9.18	Difficulties in expressing content	0.56	<0.001*
					Difficulties in receiving content	0.74	<0.001*
					I consider myself a good communicator	0.83	<0.001*
	Difficulties in expressing content	861 (51.01)	29.08	9.45	Not consider a good communicator	0.56	<0.001*
					Difficulties in receiving content	0.65	<0.001*
					I consider myself a good communicator	0.75	<0.001*
	Difficulties in receiving content	256 (15.17)	34.03	9.42	Not consider a good communicator	0.74	<0.001*
					Difficulties in expressing content	0.65	<0.001*
					I consider myself a good communicator	0.89	<0.001*
	I consider myself a good communicator	180 (10.66)	39.26	7.26	Not consider a good communicator	0.83	<0.001*
					Difficulties in expressing content	0.75	<0.001*
					Difficulties in receiving content	0.89	<0.001*
SSPS P	Not consider as a good communicator	391 (23.16)	12.80	5.10	Difficulties in expressing content	0.29	<0.001*
					Difficulties in receiving content	0.38	<0.001*
					I consider myself a good communicator	0.43	<0.001*
	Difficulties in expressing content	861 (51.01)	15.56	4.72	Not consider a good communicator	0.29	<0.001*
					Difficulties in receiving content	0.34	<0.001*
					I consider myself a good communicator	0.39	<0.001*
	Difficulties in receiving content	256 (15.17)	17.35	4.57	Not consider a good communicator	0.38	<0.001*
					Difficulties in expressing content	0.34	<0.001*
					I consider myself a good communicator	0.46	<0.001*
	I consider myself a good communicator	180 (10.66)	19.03	4.10	Not consider a good communicator	0.43	<0.001*
					Difficulties in expressing content	0.39	<0.001*
					Difficulties in receiving content	0.46	<0.001*
SSPS N	Not consider a good communicator	391 (23.16)	9.23	5.91	Difficulties in expressing content	0.37	<0.001*
					Difficulties in receiving content	0.49	<0.001*
	Difficulties in expressing content				I consider myself a good communicator	0.55	<0.001*
		861 (51.01)	13.52	6.34	Not consider a good communicator	0.37	<0.001*
					Difficulties in receiving content	0.43	<0.001*
					I consider myself a good communicator	0.49	<0.001*
	Difficulties in receiving content	256 (15.17)	16.68	6.18	Not consider a good communicator	0.49	<0.001*
					Difficulties in expressing content	0.43	<0.001*
					I consider myself a good communicator	0.59	<0.001*
	I consider myself a good communicator	180 (10.66)	20.23	4.93	Not consider a good communicator	0.55	<0.001*
					Difficulties in expressing content	0.49	<0.001*
					Difficulties in receiving content	0.59	<0.001*

**Statistics:** One-Way ANOVA, Tukey's Post-hoc

**Caption**: SSPS T = total SSPS; SSPS P = SSPS Positive Self-Perception Subscale; SSPS N = SSPS negative self-perception subscale; SD = Standard deviation; SE = Standard error.

In the pairwise crossing of the SSPS scale scores in different extracts of self-perception as a good communicator, a significant difference was evidenced in the SSPS T between those who do not consider themselves good communicators (mean of 22.03 points) with those who had difficulties in expressing information (average difference of 7.05 points, p<0.001). The difference was also seen in those with difficulty in the reception of information (average difference of 11.99 points, p<0.001) and those who considered themselves good communicators (average difference of 17.23 points, p<0.001). The same occurred in the SSPS P and SSPS N subscales, with a discrepancy between those who did not consider themselves good communicators and those who considered themselves to be good communicators (SSPS P= mean difference of 6.23 points, p<0.001; SSPS N = mean difference of 10.99 points, p<0.001).

### Ease of expressing oneself

In Table 3, the SSPS scores showed differences in the comparison of students at different levels of expressiveness: Those who (1) could not express themselves, those who (2) consider having a lot of difficulties expressing themselves, those who (3) consider having some difficulty expressing themselves, and those who (4) were able to communicate with ease. The four groups showed differences in the SSPS T (p<0.001), and in SSPS P (p<0.001), and SSPS N (p<0.001). Most participants considered expressing themselves with some difficulty (n=954, 56.5%). Students who found it easy to express themselves recorded higher scores, thus suffering less psychological impact when speaking in public.

**Table 3:** Comparisons between SSPS subscales and ease of expression in university students (n=1688)

	**Ease of expressing oneself**	**n (%)**	**Mean**	**SD**	**Relationship with another level**	**SE**	**p-value**
SSPS T	Can't express oneself	65 (3.85)	20.09	9.37	Expresses with great difficulty	1.27	0.421
					Expresses with some difficulty	1.18	<0.001*
					Expresses oneself easily	1.23	<0.001*
	Expresses with great difficulty	282 (16.70)	22.03	8.91	Can't express oneself	1.27	0.421
					Expresses with some difficulty	0.62	<0.001*
					Expresses oneself easily	0.72	<0.001*
	Expresses with some difficulty	954 (56.52)	28.96	9.49	Can't express oneself	1.18	<0.001*
					Expresses with great difficulty	0.62	<0.001*
					Expresses oneself easily	0.55	<0.001*
	Expresses oneself easily	387 (22.93)	36.91	8.69	Can't express oneself	1.23	<0.001*
					Expresses with great difficulty	0.72	<0.001*
					Expresses with some difficulty	0.55	<0.001*
SSPS P	Can't express oneself	65 (3.85)	11.97	5.21	Expresses with great difficulty	0.65	0.608
					Expresses with some difficulty	0.61	<0.001*
					Expresses oneself easily	0.63	<0.001*
	Expresses with great difficulty	282 (16.70)	12.77	5.07	Can't express oneself	0.65	0.608
					Expresses with some difficulty	0.32	<0.001*
					Expresses oneself easily	0.37	<0.001*
	Expresses with some difficulty	954 (56.52)	15.53	4.77	Can't express oneself	0.61	<0.001*
					Expresses with great difficulty	0.32	<0.001*
					Expresses oneself easily	0.28	<0.001*
	Expresses oneself easily	387 (22.93)	18.29	4.26	Can't express oneself	0.63	<0.001*
					Expresses with great difficulty	0.37	<0.001*
					Expresses with some difficulty	0.28	<0.001*
SSPS N	Can't express oneself	65 (3.85)	8.12	6.48	Expresses with great difficulty	0.84	0.532
					Expresses with some difficulty	0.78	<0.001*
	Expresses with great difficulty				Expresses oneself easily	0.82	<0.001*
		282 (16.70)	9.26	5.74	Can't express oneself	0.84	0.532
					Expresses with some difficulty	0.41	<0.001*
					Expresses oneself easily	0.48	<0.001*
	Expresses with some difficulty	954 (56.52)	13.44	6.35	Can't express oneself	0.78	<0.001*
					Expresses with great difficulty	0.41	<0.001*
					Expresses oneself easily	0.37	<0.001*
	Expresses oneself easily	387 (22.93)	18.62	5.73	Can't express oneself	0.82	<0.001*
					Expresses with great difficulty	0.48	<0.001*
					Expresses with some difficulty	0.37	<0.001*

**Statistics:** One-way ANOVA, Tukey's Post-hoc test

**Caption:** SSPS T = Total SSPS; SSPS P = SSPS Positive Self-Perception Subscale; SSPS N = SSPS negative self-perception subscale; SD = Standard Deviation; SE = Standard error.

In the cross-comparison between the SSPS scale scores and the ease of expressing themselves, the SSPS T scores of the participants who considered they could not express themselves showed a statistically significant difference in relation to the scores of those who expressed themselves with some difficulty (mean difference of 8.87 points, p<0.001) and those who expressed themselves easily (mean difference of 16.82 points, p<0.001). When comparing those who have a lot of difficulties, there is a relevant difference with students who had little (average difference of 6.93 points, p<0.001) or no difficulty (average difference of 14.88 points, p<0.001) to express themselves. These differences also occur in the SSPS P and SSPS N subscales – in which those who cannot express themselves showed marked differences when compared to those who express themselves easily (SSPS P = mean difference of 6.32 points, p<0.001; SSPS N = mean difference 10.49 points, p<0.001).

### Self-reported shyness

Table 4 shows the SSPS scores related to self-reported shyness, in which the participants considered themselves not shy, somewhat shy, or very shy. The groups differed, both in SSPS T (p<0.001), and in SSPS P (p<0.001) and SSPS N (p<0.001). Most students considered themselves a little shy (n=979, 58%). Individuals who considered themselves very shy scored lower (mean of 22.23 points), which indicates that they were more likely to feel uncomfortable when speaking to an audience when compared to the non-shy (average of 38.67).

**Table 4:** Comparisons between SSPS subscales and self-reported shyness in university students (n=1688)

	**Shyness level**	**n (%)**	**Mean**	**Standard deviation**	**Relationship with another level**	**Standard error**	**p-value**
SSPS Total	Not shy	189 (11.2)	38.67	7.38	A little shy	0.72	<0.001*
					Very shy	0.77	<0.001*
	A little shy	979 (58.0)	31.22	9.37	Not shy	0.72	<0.001*
					Very shy	0.49	<0.001*
	Very shy	520 (30.8)	22.23	9.17	Not shy	0.77	<0.001*
					A little shy	0.49	<0.001*
SSPS Positive	Not shy	189 (11.2)	18.85	4.13	A little shy	0.37	<0.001*
					Very shy	0.39	<0.001*
	A little shy	979 (58.0)	16.40	4.65	Not shy	0.37	<0.001*
					Very shy	0.25	<0.001*
	Very shy	520 (30.8)	12.79	4.91	Not shy	0.39	<0.001*
					A little shy	0.25	<0.001*
SSPS Negative	Not shy	189 (11.2)	19.83	5.03	A little shy	0.48	<0.001*
					Very shy	0.52	<0.001*
	A little shy	979 (58.0)	14.82	6.22	Not shy	0.48	<0.001*
					Very shy	0.33	<0.001*
	Very shy	520 (30.8)	9.44	6.16	Not shy	0.52	<0.001*
					A little shy	0.33	<0.001*

**Statistics:** One-Way ANOVA, Tukey's Post-hoc

### Correlations

Mostly negligible (between 0 and 0.3) and weak (between 0.3 and 0.5) correlations were found between the variables, according to the information presented in Table 5. The SSPS obtained a weak correlation with the perception as a good speaker (0.37), the ease of expressing oneself (0.47), and the degree of shyness (-0.49). The other variables of interest in the study – perception as a good speaker (GC), ease of expression (EE), and Shyness – showed weak correlations with each other: GC vs. EE (0.45); GC vs. Shyness (-0.28); EE vs. Shyness (-0.37).

**Table 5:** Correlation between variables

	Sex	Age		Income	Period	Work	GC	EE	Shyness		SSPS T	SSPS P	SSPS N
Sex	1.00												
Age	-0.09**		1.00										
Income	-0.01		0.02	1.00									
Period	0.04		0.18**	0.07**	1.00								
Work.	-0.04		0.21**	0.02	0.16**	1.00							
GC	0.01		-0.04	0.09**	-0.05**	0.08**	1.00						
EE	0.03		0.06*	0.11**	0.01	0.10**	0.45**	1.00					
Shyness	-0.00		-0.06*	-0.08**	-0.02	-0.15**	-0.28**	-0.37**	1.00				
SSPS T	-0.07**		0.07**	0.06*	0.00	0.12**	0.37**	0.47**	-0.49**	1.00			
SSPS P	-0.04		0.06*	-0.01	-0.02	0.09**	0.28**	0.36**	-0.39**	0.83**		1.00	
SSPS N	-0.07**		0.06**	0.10**	0.01	0.12**	0.35**	0.45**	-0.47**	0.91**		0.53**	1.00

**Statistics:** Pearson's Correlation Coefficient (r)

* considers p≤0.05 values significant;

** considers p≤0.01 values significant

**Caption:** GC = Consider yourself a good communicator; EE = Ease of expression; SSPS T = Self-Statements during Public Speaking, Total scale; SSPS P = Self-Statements during Public Speaking, Positive Self-Assessment Subscale; SSPS N = Self-Statements during Public Speaking, Negative Self-Assessment Subscale

## DISCUSSION

This study compared university students' self-assessment when speaking in public with the self-perception of communication aspects, such as considering themselves a good communicator, being able to express themselves easily, and self-assessment related to shyness. Most individuals were found to have difficulties in expressing content (51.0%), with some difficulty in expressing themselves (56.5%) and somewhat shy (58.0%).

The average found on the SSPS scale was 29.28 points - which corresponds to 58.56% of the total possible score in this assessment tool, which is considered a level of favorable perception of self-assessment when speaking in public. In a study with the same public – university students – the average was 25.96 points^(5)^. These data showed that Brazilian university students have an intermediate self-perception regarding their ability to speak in public. When analyzing the positive and negative self-perception subscales, the means were 15.56 and 13.72 points, respectively. In the literature, mean values of positive self-perception are similar to those found in this study: 16.73 points for Brazilian university students^(5)^, 15.8 points for American university students^(14)^, and 15.15 points for adolescents^(17)^.

In the negative self-perception subscale, the values found in the literature were: 9.34 points for Brazilian university students^(5)^, 7.1 points for American university students^(14)^, and 5.47 points for adolescents^(16)^. This subscale is related to negative effects and depression, being more sensitive to detecting individuals with social anxiety disorders^(14,15)^. When comparing the two subscales, the positive self-perception scores were higher when compared to the negative subscale, in line with other studies^(5,14,17)^.

In a study with different professional groups, the SSPS T scale scores presented averages between 37.8 points - in technical support professionals, and 39.7 points – in informing-type professionals. The support professionals have long periods of voice use, with frequent periods of silence, and often deal with stress. The informing-type professionals tend to work with uninterrupted use of voice, varying the number of people and the size of the space where they speak^(18)^. These values are higher in relation to the values obtained in the present study. This difference is due to the different levels of experience between the group of students and professionals, as university students are still developing their public speaking skills, while professionals routinely exercise these communication skills.

Regarding self-perception as a communicator, 180 (10.6%) respondents considered themselves to be good communicators. There was also a difference of 17 points in the SSPS between those who considered themselves to be good communicators compared to those who did not, with greater difficulties in expressing (speaking) than in receiving (listening) content. When analyzing the perception of ease in expressing themselves, only 387 (22.9%) did not report difficulties. Even in fluent speakers, changes in communication can be manifested – such as hesitations and the occurrence of occasional disfluencies^(19)^. Communication skills are essential for the effective practice of any profession, and the development of these skills in the academic context is highly encouraged. Communication competence requires skills of persuasion, information, speaking, listening, and interpersonal relationships^(3)^. In addition, proximity and involvement with the public, mastery of the content, clarity, good communication skills, and empathy are also considered important public speaking characteristics for a good perception from the audience^(20)^. Sánchez Expósito et al.^(21)^ described in their research with nursing students that they focused mainly on clinical skills and not so much on communication skills with patients, suggesting the training of these skills during professional training. In comparison to the aforementioned study, an Australian survey^(22)^ pointed out that its sample of students showed approximately 70% confidence in their communication skills at the beginning of graduation, with the group reaching levels of 75% at the end of the undergraduate period. This study demonstrated the importance of mapping academic activities that promote the use of communication throughout undergraduate studies, to better prepare this student for the job market.

Regarding shyness, there was a predominance of participants who rated themselves as being “a little shy”. As a consequence of shyness, such individuals take less advantage of social situations, date less, feel more lonely, and are less expressive, both verbally and non-verbally^(23)^. Shy people potentially find it more difficult to get a job, as the job market increasingly requires individuals to have good interpersonal skills^(23)^. According to a Brazilian study^(24)^ carried out with university students, participants who considered themselves shy were more likely to be afraid of public speaking, participating little in activities that involved public communication. They also presented negative self-assessment of speech, low vocal intensity, elevated speaking speed, and poor visual contact with the audience. In a multivariate model analysis, participants who claimed to be afraid of public speaking and who had low vocal intensity were more likely to perceive themselves as shy. Such data are in line with the findings of this study, in which participants who considered themselves very shy had lower averages of self-perception when speaking in public.

Data collection in the virtual mode may have been a limitation to the present research, since the participants answered according to their interpretations of the questions, without support or interference from the researchers, if necessary.

Data from this study are based specifically on participant’ self-assessments. Therefore, clinical studies are suggested to assess whether interventions involving communication enhancement influence self-perception in public speaking situations.

## CONCLUSION

The present study compared public speaking self-assessment (SSPS) with self-perception of communication skills and shyness. Participants with better communicational self-perception and lower self-perception of shyness presented a more favorable self-assessment regarding public presentations.
