# Supplementary material for: Impacts of self-reported communication perception and shyness on the public speaking assessment of university students
Source: Codas. 2022 Oct 17;35(1):e20210225. doi: 10.1590/2317-1782/20212021225en (PMC10010429; doi:10.1590/2317-1782/20212021225en)
Supplement: Translation

# Translation: Impactos da percepção comunicacional e da timidez autorreferidos na avaliação ao falar em público de estudantes universitários

**DOI:** 10.1590/2317-1782/20212021225pt

**Authors:** Daniel Lucas Picanço Marchand, Lucas Sávio Rodrigues Carvalho, Diego de Souza Leal, Sheila Gonçalves Câmara, Glaucya Madazio, Mara Behlau, Mauriceia Cassol

**Affiliations:** 1 Universidade Federal de Ciências da Saúde de Porto Alegre – UFCSPA - Porto Alegre (RS), Brasil.; 2 Centro de Estudos da Voz – CEV - São Paulo (SP), Brasil.; 3 Escola de Saúde Pública do Rio Grande do Sul – ESP/RS - Porto Alegre (RS), Brasil.

**Keywords:** Fonoaudiologia, Fala, Comunicação, Barreiras de Comunicação, Timidez, Comunicação Persuasiva, Relações Interpessoais

## Abstract

**Objetivo:**

comparar a autoavaliação ao falar em público, por meio da Escala para Autoavaliação ao Falar em Público, com a percepção comunicacional e timidez autorreferidos, de estudantes universitários.

**Método:**

tratou-se de um estudo observacional transversal prospectivo. Foram convidados a participar deste estudo, estudantes universitários do Brasil de diferentes áreas de conhecimento, sendo incluídos os que assentiram em participar. Os participantes receberam convite eletrônico e preencheram um formulário elaborado na plataforma Google Forms que continha questões sociodemográficas, sobre autopercepção como bom falante, sobre facilidade para se expressar, sobre timidez, e a Escala para Autoavaliação ao Falar em Público. Foram comparadas as médias da Escala de Autoavaliação ao Falar em Público com a autopercepção como bom falante, com a facilidade para se expressar e com a timidez.

**Resultados:**

os participantes que se consideravam bons comunicadores, os que acreditavam ter facilidade para se expressar e os que não eram tímidos apresentaram melhor autopercepção sobre as suas habilidades de fala em público.

**Conclusão:**

a autopercepção comunicacional positiva, bem como a autopercepção de menor timidez estão relacionadas a uma autoavaliação mais favorável em relação a apresentações em público.

## INTRODUÇÃO

A comunicação permeia as relações interpessoais e depende não só de habilidades linguísticas, como também sofre influência dos aspectos psicológicos de um sujeito^(1)^. No contexto acadêmico, uma importante forma de comunicação exercitada são as apresentações orais^(2)^ sob a forma de fala dos universitários, tendo seus pares como público. Essa aptidão é cada vez mais exigida, uma vez que futuramente será crucial para esses graduandos em seus diversos ambientes de trabalho^(3)^.

O medo de falar em público, considerado um subtipo de ansiedade social, atinge uma parcela considerável da população, com alta prevalência em estudantes universitários^(4,5)^. Costuma estar associado à apreensão comunicacional, caracterizada como um tipo de timidez relacionada à expectativa de falar para pessoas^(6)^. Ambos afetam a habilidade de criar e decifrar mensagens, além de incitar uma autoavaliação negativa, podendo impactar as esferas pessoal e profissional de um indivíduo^(4,7)^.

A habilidade de falar bem em público promove satisfação tanto social, quanto na autoestima de um sujeito^(8)^. Contudo, a preocupação de ser julgado, a comparação de si com outros, além da antecipação de prejuízos decorrentes de uma apresentação oral insatisfatória tornam a experiência de falar em público desconfortável^(8)^. Assim, acabam sofrendo com medo de falar em público os indivíduos com falta de confiança, timidez, preparação inadequada, tensão, medo de errar e pobres habilidade de fala no idioma^(9)^. A timidez tem um papel especial nessas dificuldades de enfrentar a situação de falar em público, por ser uma autoavaliação negativa antecipatória. A timidez é considerada um traço de personalidade na qual um indivíduo faz avaliações negativas de si, ao criar desconfortos ou inibições em situações sociais, potencialmente produzindo barreiras no trabalho, nas amizades, nas relações afetivas e no lazer^(10,11)^.

Visando compreender as causas e impactos do medo de falar em público em um indivíduo, alguns protocolos e escalas foram criados^(12-14)^. Uma dessas escalas é a *Self-Statements during Public Speaking* – SSPS^(14)^, com adaptação transcultural^(15)^ e validação psicométrica para o português brasileiro^(16)^, passando a ser chamada de Escala para Autoavaliação ao Falar em Público. O objetivo da ferramenta é avaliar pensamentos temerosos durante uma apresentação para uma plateia.

De acordo com a literatura, o estado emocional influencia fatores comunicacionais. Assim, o objetivo deste estudo é comparar a autoavaliação ao falar em público, por meio da escala SSPS, com a percepção comunicacional e timidez autorreferidos, de estudantes universitários.

## MÉTODO

### Desenho da pesquisa

Trata-se de um estudo observacional transversal prospectivo aprovado pelo Comitê de Ética em Pesquisa da instituição proponente sob parecer 2.729.273. Devido ao desenho de estudo, foi utilizado o *checklist* STROBE a fim de nortear a escrita.

### Participantes

Foram convidados a participar do estudo estudantes de graduação que frequentassem instituições de Ensino Superior no Brasil. Foram incluídos todos os participantes que assentiram em responder as avaliações, concordando com o disposto no Termo de Consentimento Livre e Esclarecido, e excluídos aqueles que possuíam idade inferior a 18 anos ou que forneceram respostas incorretas e/ou incompletas aos instrumentos de avaliação.

### Cálculo amostral

Realizou-se um estudo piloto utilizando as respostas de 30 participantes escolhidos aleatoriamente. Com base nesse estudo e considerando a população de estudantes heterogênea quanto às respostas (50%), erro amostral de 5% e confiança de 95%, a quantidade mínima de respostas para a amostra ser representativa dessa população foi estimada em 384. Esperou-se, ainda, uma taxa de resposta de 30% - dessa forma, necessitou-se convidar pelo menos 1280 estudantes a responderem à pesquisa.

### Coleta de dados

Os participantes receberam convite, via e-mail ou redes sociais, contendo um formulário eletrônico construído na plataforma *Google Forms*. Foi solicitado que respondessem a perguntas gerais de identificação, de comunicação, e a Escala de Autoavaliação ao Falar em Público (SSPS). A coleta ocorreu entre outubro de 2018 e junho de 2020.

#### Questionário sobre comunicação

Foi solicitado aos participantes que respondessem às seguintes questões fechadas, criadas pelos autores (Quadro 1).

**Quadro 1:** Questionário sobre comunicação

**Você se considera um bom comunicador?**
*Entenda como um bom comunicador: uma pessoa que consegue expressar suas ideias verbalmente com facilidade e prestar atenção àquilo que estão lhe dizendo*

() Definitivamente me considero um bom comunicador
() Às vezes consigo ser um bom comunicador, pois consigo me expressar bem. Porém ocasionalmente não presto atenção ao conteúdo da mensagem
() Às vezes consigo ser um bom comunicador, pois presto atenção naquilo que me dizem. Porém ocasionalmente tenho dificuldades para me expressar
() Não me considero um bom comunicador
**Quando você precisa se comunicar, você:**
() Consegue se expressar com muita facilidade, sem a necessidade de complementar ou repetir o que foi dito
() Consegue se expressar com alguma dificuldade, muitas vezes precisando complementar ou repetir o que foi dito
() Consegue se expressar com muita dificuldade, quase sempre precisando complementar ou repetir o que foi dito
() Não consegue se expressar, sempre precisando complementar ou repetir o que foi dito
**Quanto à timidez, você se considera:**
() Não tímido
() Um pouco tímido
() Muito tímido

#### Escala de Autoavaliação ao Falar em Público (SSPS)

A SSPS^(14-16)^ apresenta 10 questões em que o participante deve responder tendo em mente a sua percepção em situações de fala em público. A chave de respostas corresponde a uma escala do tipo *Likert* de seis pontos, sendo marcado 0 quando o respondente discordar totalmente da afirmação e 5, quando concordar totalmente. A escala apresenta duas subescalas de cinco questões cada: autoavaliação positiva (SSPS P), e autoavaliação negativa (SSPS N). O escore total (SSPS T) se dá pelo somatório da pontuação das 10 questões, sendo invertida a pontuação das afirmativas da subescala de autoavaliação negativa. O escore final dessa escala é entre 0 e 50 pontos e a interpretação do resultado é: quanto maior a pontuação, mais à vontade ao falar em público um sujeito se sente.

### Amostragem

Após a aplicação dos critérios de elegibilidade (Figura 1), a amostra final é composta por 1688 participantes.

**Figura 1:** Fluxograma de composição da amostra

A Tabela 1 dispõe dos dados de caracterização da amostra. A amostra é composta por participantes com idade entre 18 e 61 anos (24,00 e 5,74 - média e desvio padrão, respectivamente), sobretudo por mulheres (n=1103, 65,3%), caucasianos (n=827, 49%) e estudantes de universidade pública (n=1555, 92,1%). A área de conhecimento dos participantes e a região geográfica não apresentaram diferença estatística em relação às variáveis analisadas neste estudo.

**Tabela 1:** Características da amostra de estudantes universitários (n=1688)

**Variável**		**Descrição**
Sexo – n (%)	Masculino	585 (34,7)
	Feminino	1103 (65,3)
Idade – $\bar{X}$ ± DP		24,00 ± 5,74
Etnia – n (%)	Branca	827 (49,0)
	Negra	234 (13,9)
	Parda	584 (34,6)
	Amarela	37 (2,2)
	Indígena	6 (0,4)
Tipo de faculdade – n (%)	Pública	1555 (92,1)
	Privada	133 (7,9)
Região – n (%)	Norte	61 (3,6)
	Nordeste	552 (32,7)
	Centro-Oeste	82 (4,9)
	Sudeste	607 (36,0)
	Sul	383 (22,7)
	Outro país	3 (0,1)
Área de conhecimento – n (%)	Agrárias	177 (10,2)
	Biológicas	90 (5,3)
	Saúde	392 (23,2)
	Exatas	218 (12,9)
	Humanas	172 (10,2)
	Sociais	420 (24,9)
	Engenharias	158 (9,4)
	Linguística	66 (3,9)
Anos de graduação – n (%)	1º ano	305 (18,1)
	2º ano	406 (24,1)
	3º ano	345 (20,4)
	4º ano	380 (22,5)
	5º ano	208 (12,3)
	6 ou mais anos	44 (2,6)
Renda – n (%)	Menos de 1 salário mínimo (R$0,00 ~ R$953,99)	276 (16,4)
	De 1 a 3 salários mínimos (R$954,00 ~ R$2861,99)	698 (41,4)
	De 3 a 6 salários mínimos (R$2862,00 ~ R$5723,99)	393 (23,3)
	De 6 a 10 salários mínimos (R$5724,00 ~ R$9539,99)	201 (11,9)
	Mais de 10 salários mínimos (R$9540,00 ou mais)	120 (7,1)
Trabalha – n (%)	Não	972 (57,6)
	Sim	716 (42,4)

**Legenda**: $\bar{X}$ = Média; DP = Desvio-padrão.

### Tratamento estatístico

A análise da normalidade de distribuição dos dados foi aferida por meio do teste de Kolmogorov-Smirnov. As variáveis categóricas foram expressas por valores absolutos e relativos, enquanto que as variáveis quantitativas, por média e desvio padrão. Foi utilizada a Análise de Variância (ANOVA) de uma via para comparar os escores na escala SSPS com as demais variáveis, e o post-hoc de Tukey para identificar em quais categorias houve significância. Para a análise de correlação entre variáveis foi aplicado o Coeficiente de Correlação de Pearson. Adotou-se como nível de significância p≤0,05. As análises estatísticas foram realizadas com o *software* Statistical Package for Social Sciences (SPSS) versão 25.0 (IBM Corp. Released 2017. IBM SPSS Statistics for Windows. IBM Corp., Armonk, NY).

## RESULTADOS

### Percepção como comunicador

A Tabela 2 mostrou que os escores do SSPS foram diferentes ao se comparar os estudantes que se autoavaliaram como: (1) Bons comunicadores, aqueles que (2) apresentavam dificuldade na expressão, aqueles que (3) apresentavam dificuldades na recepção da informação e, ainda, aqueles que (4) não se consideraram bons comunicadores. Os resultados dos quatro grupos foram diferentes, tanto no SSPS T (p<0,001), como no SSPS P (p<0,001) e SSPS N (p<0,001). As médias da amostra foram de 29,28 ± 10,50 para o SSPS T, 15,56 ± 5,08 para o SSPS P e 13,72 ± 6,89 para o SSPS N. A amostra contou, sobretudo, com estudantes que consideravam ter dificuldades na expressão de conteúdo (n=861, 51%). Os bons comunicadores pontuaram mais alto, ou seja, sentem-se mais confortáveis para falar em público, seguidos por aqueles que tem dificuldade na recepção, depois na expressão e, por fim, os que não se consideraram bons comunicadores.

**Tabela 2:** Comparações entre as subescalas do SSPS e autopercepção como bom comunicador em estudantes universitários (n=1688)

	Autopercepção como bom comunicador	n (%)	Média	DP	Relação com outro nível	EP	p-valor
SSPS T	Não me considero bom comunicador	391 (23,16)	22,03	9,18	Dificuldades na expressão de conteúdo	0,56	<0,001*
					Dificuldades na recepção de conteúdo	0,74	<0,001*
					Considero-me um bom comunicador	0,83	<0,001*
	Dificuldades na expressão de conteúdo	861 (51,01)	29,08	9.45	Não me considero bom comunicador	0,56	<0,001*
					Dificuldades na recepção de conteúdo	0,65	<0,001*
					Considero-me um bom comunicador	0,75	<0,001*
	Dificuldades na recepção de conteúdo	256 (15,17)	34,03	9,42	Não me considero bom comunicador	0,74	<0,001*
					Dificuldades na expressão de conteúdo	0,65	<0,001*
					Considero-me um bom comunicador	0,89	<0,001*
	Considero-me um bom comunicador	180 (10,66)	39,26	7,26	Não me considero bom comunicador	0,83	<0,001*
					Dificuldades na expressão de conteúdo	0,75	<0,001*
					Dificuldades na recepção de conteúdo	0,89	<0,001*
SSPS P	Não me considero bom comunicador	391 (23,16)	12,80	5,10	Dificuldades na expressão de conteúdo	0,29	<0,001*
					Dificuldades na recepção de conteúdo	0,38	<0,001*
					Considero-me um bom comunicador	0,43	<0,001*
	Dificuldades na expressão de conteúdo	861 (51,01)	15,56	4,72	Não me considero bom comunicador	0,29	<0,001*
					Dificuldades na recepção de conteúdo	0,34	<0,001*
					Considero-me um bom comunicador	0,39	<0,001*
	Dificuldades na recepção de conteúdo	256 (15,17)	17,35	4,57	Não me considero bom comunicador	0,38	<0,001*
					Dificuldades na expressão de conteúdo	0,34	<0,001*
					Considero-me um bom comunicador	0,46	<0,001*
	Considero-me um bom comunicador	180 (10,66)	19,03	4,10	Não me considero bom comunicador	0,43	<0,001*
					Dificuldades na expressão de conteúdo	0,39	<0,001*
					Dificuldades na recepção de conteúdo	0,46	<0,001*
SSPS N	Não me considero bom comunicador	391 (23,16)	9,23	5,91	Dificuldades na expressão de conteúdo	0,37	<0,001*
					Dificuldades na recepção de conteúdo	0,49	<0,001*
	Dificuldades na expressão de conteúdo				Considero-me um bom comunicador	0,55	<0,001*
		861 (51,01)	13,52	6,34	Não me considero bom comunicador	0,37	<0,001*
					Dificuldades na recepção de conteúdo	0,43	<0,001*
					Considero-me um bom comunicador	0,49	<0,001*
	Dificuldades na recepção de conteúdo	256 (15,17)	16,68	6,18	Não me considero bom comunicador	0,49	<0,001*
					Dificuldades na expressão de conteúdo	0,43	<0,001*
					Considero-me um bom comunicador	0,59	<0,001*
	Considero-me um bom comunicador	180 (10,66)	20,23	4,93	Não me considero bom comunicador	0,55	<0,001*
					Dificuldades na expressão de conteúdo	0,49	<0,001*
					Dificuldades na recepção de conteúdo	0,59	<0,001*

**Estatística**: Teste ANOVA de uma via, Post-hoc de Tukey

**Legenda**: SSPS T = SSPS total; SSPS P = SSPS subescala de autopercepção positiva; SSPS N = SSPS subescala de autopercepção negativa; DP = Desvio padrão; EP = Erro padrão.

No cruzamento por pares dos escores da escala SSPS em diferentes extratos de autopercepção como bom comunicador evidenciou-se na SSPS T uma diferença significativa entre aqueles que não se consideram bons comunicadores (média de 22,03 pontos) com aqueles que tinham dificuldades na expressão da informação (diferença média de 7,05 pontos, p<0,001), na recepção da informação (diferença média de 11,99 pontos, p<0,001) e aqueles que se consideravam bons comunicadores (diferença média de 17,23 pontos, p<0,001). O mesmo ocorreu nas subescalas SSPS P e SSPS N, havendo discrepância entre os que não se consideravam bons comunicadores e os que se consideravam (SSPS P= diferença média de 6,23 pontos, p<0,001; SSPS N = diferença média de 10,99 pontos, p<0,001).

### Facilidade de se expressar

Na Tabela 3 os escores do SSPS apresentaram diferenças na comparação dos estudantes em distintos níveis de expressividade: Aqueles que (1) não conseguiam se expressar, aqueles que (2) possuíam muita dificuldade para se expressar, aqueles que (3) possuíam alguma dificuldade para se expressar e, por fim, aqueles que (4) possuíam facilidade para se comunicar. Os quatro grupos apresentaram diferenças, tanto no SSPS T (p<0,001), como no SSPS P (p<0,001) e SSPS N (p<0,001). A maioria dos participantes consideravam se expressar com alguma dificuldade (n=954, 56,5%). Os estudantes que consideravam ter facilidade para se expressar registraram maiores pontuações, desta forma sofrem menor impacto psicológico ao falar em público.

**Tabela 3:** Comparações entre as subescalas do SSPS e facilidade para se expressar em estudantes universitários (n=1688)

	Facilidade para se expressar	n (%)	Média	DP	Relação com outro nível	EP	**p-valor**
SSPS T	Não consegue se expressar	65 (3,85)	20,09	9,37	Expressa-se com muita dificuldade	1,27	0,421
					Expressa-se com alguma dificuldade	1,18	<0,001*
					Expressa-se com facilidade	1,23	<0,001*
	Expressa-se com muita dificuldade	282 (16,70)	22,03	8,91	Não consegue se expressar	1,27	0,421
					Expressa-se com alguma dificuldade	0,62	<0,001*
					Expressa-se com facilidade	0,72	<0,001*
	Expressa-se com alguma dificuldade	954 (56,52)	28,96	9,49	Não consegue se expressar	1,18	<0,001*
					Expressa-se com muita dificuldade	0,62	<0,001*
					Expressa-se com facilidade	0,55	<0,001*
	Expressa-se com facilidade	387 (22,93)	36,91	8,69	Não consegue se expressar	1,23	<0,001*
					Expressa-se com muita dificuldade	0,72	<0,001*
					Expressa-se com alguma dificuldade	0,55	<0,001*
SSPS P	Não consegue se expressar	65 (3,85)	11,97	5,21	Expressa-se com muita dificuldade	0,65	0,608
					Expressa-se com alguma dificuldade	0,61	<0,001*
					Expressa-se com facilidade	0,63	<0,001*
	Expressa-se com muita dificuldade	282 (16,70)	12,77	5,07	Não consegue se expressar	0,65	0,608
					Expressa-se com alguma dificuldade	0,32	<0,001*
					Expressa-se com facilidade	0,37	<0,001*
	Expressa-se com alguma dificuldade	954 (56,52)	15,53	4,77	Não consegue se expressar	0,61	<0,001*
					Expressa-se com muita dificuldade	0,32	<0,001*
					Expressa-se com facilidade	0,28	<0,001*
	Expressa-se com facilidade	387 (22,93)	18,29	4,26	Não consegue se expressar	0,63	<0,001*
					Expressa-se com muita dificuldade	0,37	<0,001*
					Expressa-se com alguma dificuldade	0,28	<0,001*
SSPS N	Não consegue se expressar	65 (3,85)	8,12	6,48	Expressa-se com muita dificuldade	0,84	0,532
					Expressa-se com alguma dificuldade	0,78	<0,001*
	Expressa-se com muita dificuldade				Expressa-se com facilidade	0,82	<0,001*
		282 (16,70)	9,26	5,74	Não consegue se expressar	0,84	0,532
					Expressa-se com alguma dificuldade	0,41	<0,001*
					Expressa-se com facilidade	0,48	<0,001*
	Expressa-se com alguma dificuldade	954 (56,52)	13,44	6,35	Não consegue se expressar	0,78	<0,001*
					Expressa-se com muita dificuldade	0,41	<0,001*
					Expressa-se com facilidade	0,37	<0,001*
	Expressa-se com facilidade	387 (22,93)	18,62	5,73	Não consegue se expressar	0,82	<0,001*
					Expressa-se com muita dificuldade	0,48	<0,001*
					Expressa-se com alguma dificuldade	0,37	<0,001*

**Estatística**: Teste ANOVA de uma via, Post-hoc de Tukey

**Legenda**: SSPS T = SSPS total; SSPS P = SSPS subescala de autopercepção positiva; SSPS N = SSPS subescala de autopercepção negativa; DP = Desvio padrão; EP = Erro padrão.

Na comparação cruzada entre os escores da escala SSPS e a facilidade de se expressar os escores do SSPS T dos participantes que consideravam não conseguirem se expressar apresentaram diferença estatisticamente significativa em relação aos escores daqueles que se expressavam com alguma dificuldade (diferença média de 8,87 pontos, p<0,001) e daqueles que se expressavam com facilidade (diferença média de 16,82 pontos, p<0,001). Já quando comparados aqueles que apresentam muita dificuldade, destaca-se a diferença relevante com os estudantes que apresentavam pouca (diferença média de 6,93 pontos, p<0,001) ou nenhuma dificuldade (diferença média de 14,88 pontos, p<0,001) de se expressar. Essas diferenças também ocorrem nas subescalas SSPS P e SSPS N – em que aqueles que não conseguem se expressar apresentaram diferenças marcantes quando equiparados àqueles que se expressam com facilidade (SSPS P = diferença média de 6,32 pontos, p<0,001; SSPS N = diferença média 10,49 pontos, p<0,001).

### Timidez autorreferida

A Tabela 4 expõe os escores do SSPS relacionados à timidez autorreferida, na qual os participantes julgavam-se não-tímidos, um pouco tímidos ou muito tímidos. Os grupos se diferenciaram, tanto no SSPS T (p<0,001), como no SSPS P (p<0,001) e SSPS N (p<0,001). A maior parcela dos estudantes se considerava um pouco tímidos (n=979, 58%). Os indivíduos que se consideraram muito tímidos pontuaram mais baixo (média de 22,23 pontos), o que indica que apresentavam maiores chances de se sentirem desconfortáveis ao falarem para uma plateia quando comparados aos não tímidos (média de 38,67).

**Tabela 4:** Comparações entre as subescalas do SSPS e timidez autorreferida em estudantes universitários (n=1688)

	Nível de timidez	n (%)	Média	Desvio Padrão	Relação com outro nível	Erro padrão	Valor de p
SSPS Total	Não tímido	189 (11,2)	38,67	7,38	Um pouco tímido	0,72	<0,001*
					Muito tímido	0,77	<0,001*
	Um pouco tímido	979 (58,0)	31,22	9,37	Não tímido	0,72	<0,001*
					Muito tímido	0,49	<0,001*
	Muito tímido	520 (30,8)	22,23	9,17	Não tímido	0,77	<0,001*
					Um pouco tímido	0,49	<0,001*
SSPS Positiva	Não tímido	189 (11,2)	18,85	4,13	Um pouco tímido	0,37	<0,001*
					Muito tímido	0,39	<0,001*
	Um pouco tímido	979 (58,0)	16,40	4,65	Não tímido	0,37	<0,001*
					Muito tímido	0,25	<0,001*
	Muito tímido	520 (30,8)	12,79	4,91	Não tímido	0,39	<0,001*
					Um pouco tímido	0,25	<0,001*
SSPS Negativa	Não tímido	189 (11,2)	19,83	5,03	Um pouco tímido	0,48	<0,001*
					Muito tímido	0,52	<0,001*
	Um pouco tímido	979 (58,0)	14,82	6,22	Não tímido	0,48	<0,001*
					Muito tímido	0,33	<0,001*
	Muito tímido	520 (30,8)	9,44	6,16	Não tímido	0,52	<0,001*
					Um pouco tímido	0,33	<0,001*

**Estatística**: Teste ANOVA de uma via, Post-hoc de Tukey

### Correlações

Foram encontradas majoritariamente correlações desprezíveis (entre 0 e 0,3) e fracas (entre 0,3 e 0,5) entre as variáveis, conforme informações apresentadas na Tabela 5. A SSPS obteve correlação fraca com a percepção como bom falante (0,37), com a facilidade para se expressar (0,47), e com o grau de timidez (-0,49). As demais variáveis de interesse do estudo – percepção como bom falante (BC), facilidade para se expressar (FE) e Timidez – apresentaram correlações fracas entre si: BC *vs*. FE (0,45); BC *vs*. Timidez (-0,28); FE *vs*. Timidez (-0,37).

**Tabela 5:** Correlação entre variáveis

	Sexo	Idade	Renda	Período	Trab.	BC	FE	Timidez		SSPS T	SSPS P	SSPS N
Sexo	1,00											
Idade	-0,09^**^	1,00										
Renda	-0,01	0,02	1,00									
Período	0,04	0,18**	0,07**	1,00								
Trab.	-0,04	0,21**	0,02	0,16**	1,00							
BC	0,01	-0,04	0,09**	-0,05**	0,08**	1,00						
FE	0,03	0,06^*^	0,11**	0,01	0,10**	0,45**	1,00					
Timidez	-0,00	-0,06*	-0,08**	-0,02	-0,15**	-0,28**	-0,37**	1,00				
SSPS T	-0,07**	0,07**	0,06*	0,00	0,12**	0,37**	0,47**	-0,49**	1,00			
SSPS P	-0,04	0,06*	-0,01	-0,02	0,09**	0,28**	0,36**	-0,39**	0,83**		1,00	
SSPS N	-0,07**	0,06**	0,10**	0,01	0,12**	0,35**	0,45**	-0,47**	0,91**		0,53**	1,00

**Estatística:** Coeficiente de Correlação de Pearson (r)

* considera significativo valores de p≤0,05;

** considera significativo valores de p≤0,01

**Legenda:** Trab: Se exerce atividade remunerada; BC: Considera-se bom comunicador; FE: Facilidade para se expressar; SSPS T: Self-Statements during Public Speaking, escala Total; SSPS P: Self-Statements during Public Speaking, subescala de autoavaliação positiva; SSPS N: Self-Statements during Public Speaking, subescala de autoavaliação negativa.

## DISCUSSÃO

Este estudo comparou a autoavaliação ao falar em público de estudantes universitários com a autopercepção de aspectos comunicacionais, como considerar-se um bom comunicador, ter facilidade para se expressar, e a autoavaliação relacionada à timidez. Encontrou-se majoritariamente indivíduos com dificuldades de expressão de conteúdo (51,0%), com alguma dificuldade para se expressar (56,5%) e um pouco tímidos (58,0%).

A média encontrada na escala SSPS foi de 29,28 pontos – o que corresponde a 58,56% do escore total possível nesta ferramenta de avaliação, sendo considerado um nível de percepção favorável de autoavaliação ao falar em público. Em um estudo com o mesmo público – estudantes universitários - a média foi de 25,96 pontos^(5)^. Esses dados apontaram que estudantes universitários brasileiros apresentam uma autopercepção intermediária em relação à sua capacidade de falar em público. Ao analisar as subescalas de autopercepção positiva e negativa, as médias foram de 15,56 e 13,72 pontos, respectivamente. Na literatura os valores médios de autopercepção positiva são semelhantes aos encontrados neste estudo: 16,73 pontos em estudantes universitários brasileiros^(5)^, 15,8 pontos em estudantes universitárias americanas^(14)^ e 15,15 pontos em adolescentes^(17)^.

Na subescala de autopercepção negativa, os valores encontrados na literatura foram de: 9,34 pontos em estudantes universitários brasileiros^(5)^, 7,1 pontos em estudantes universitárias americanas^(14)^ e 5,47 pontos em adolescentes^(16)^. Essa subescala está relacionada a efeitos negativos e depressão, sendo mais sensível para detectar indivíduos com distúrbios de ansiedade social^(14,15)^. Ao analisar comparativamente as duas subescalas, os escores de autopercepção positiva foram maiores em relação aos da subescala negativa, indo ao encontro de outros estudos^(5,14,17)^.

Em estudo com diferentes grupos profissionais, os escores da escala SSPS T apresentaram médias entre 37,8 pontos – em profissionais de suporte técnico, que apresentam longos períodos de uso de voz, com frequentes períodos de silêncio, por vezes lidando com estresse – e 39,7 pontos – em profissionais informantes, que trabalham com uso ininterrupto de voz, variando a quantidade de pessoas e o tamanho do espaço onde falam^(18)^. Esses valores são mais elevados em relação aos valores obtidos no presente estudo. Essa diferença ocorre devido aos diferentes níveis de experiência entre o grupo de estudantes e profissionais, visto que os universitários ainda estão desenvolvendo suas habilidades de fala em público, enquanto os profissionais exercitam essas aptidões comunicativas rotineiramente.

Sobre a autopercepção como comunicador, 180 (10,6%) respondentes consideravam-se bons comunicadores. Houve ainda uma diferença de 17 pontos na SSPS entre aqueles que se consideram bons comunicadores em relação àqueles que não se julgaram dessa forma, havendo maiores dificuldades na expressão (fala) do que na recepção (escuta) de conteúdo. Quando analisada a percepção de facilidade para se expressar, apenas 387 (22,9%) não referiram dificuldades. Mesmo em falantes fluentes podem-se manifestar alterações na comunicação – como hesitações e ocorrência de disfluências ocasionais^(19)^. As habilidades comunicacionais são indispensáveis para a prática efetiva de qualquer profissão, sendo altamente incentivado o desenvolvimento dessas competências no contexto acadêmico. A competência comunicacional exige capacidades de persuasão, informação, fala, escuta e relação interpessoal^(3)^. Além disso, na fala em público consideram-se, ainda, a proximidade e envolvimento com o público, domínio do conteúdo, clareza, boas habilidades comunicacionais e empatia como características importantes para uma boa percepção da audiência^(20)^. Sánchez Expósito et al.^(21)^ descreveram em sua pesquisa com estudantes de enfermagem que estes se focavam sobretudo nas habilidades clínicas e nem tanto nas habilidades comunicacionais frente a um paciente, sugerindo o treinamento destas aptidões durante a formação profissional. Em comparação ao estudo citado, uma pesquisa australiana^(22)^ apontou que a sua amostra de estudantes apresentou aproximadamente 70% de confiança em suas habilidades de comunicação no começo da graduação, sendo que o grupo alcançou níveis de 75% ao final do período da graduação. Esse estudo demonstrou a importância de mapear as atividades acadêmicas que promovam o uso da comunicação ao longo da graduação, a fim de melhor preparar esse estudante para o mercado de trabalho.

Com relação à timidez houve um predomínio de participantes que se avaliaram como sendo “um pouco tímidos”. Como consequências da timidez, tais indivíduos tiram menos vantagens de situações sociais, namoram menos, sentem-se mais solitários e são menos expressivos, tanto verbalmente, quanto não-verbalmente^(23)^. Potencialmente pessoas tímidas tem mais dificuldades de conseguir emprego, visto que o mercado de trabalho cada vez mais exige que indivíduos tenham boas habilidades interpessoais^(23)^. Segundo um estudo brasileiro^(24)^ realizado com estudantes universitários, participantes que se consideravam tímidos tinham mais tendências a terem medo de falar em público, participando pouco de atividades que envolviam comunicação em público. Também apresentaram autoavaliação negativa da fala, fraca intensidade vocal, velocidade elevada de discurso e pobre contato visual com a plateia. Em uma análise de modelo multivariado, participantes que alegaram ter medo de falar em público e que tinham fraca intensidade vocal tinham maiores chances de se perceberem como tímidos. Tais dados vão ao encontro dos achados deste estudo, no qual os participantes que se consideraram muito tímidos tinham médias inferiores de autopercepção ao falar em público.

A coleta de dados na modalidade virtual pode ter sido uma limitação à presente pesquisa, visto que os participantes respondiam conforme as suas interpretações das perguntas, sem suporte ou interferência dos pesquisadores, caso necessitassem.

Os dados deste estudo são baseados especificamente em autoavaliações de participantes. Sendo assim, sugere-se estudos clínicos visando avaliar se intervenções que envolvam aprimoramento comunicacional influenciam na autopercepção em situações de fala em público.

## CONCLUSÃO

O presente estudo comparou a autoavaliação ao falar em público (SSPS) com a autopercepção sobre habilidades de comunicação e timidez. Os participantes com melhor autopercepção comunicacional e menor autopercepção de timidez apresentaram uma autoavaliação mais favorável em relação a apresentações em público.
